# Possible Synergistic Effects of Thymol and Nicotine against *Crithidia bombi* Parasitism in Bumble Bees

**DOI:** 10.1371/journal.pone.0144668

**Published:** 2015-12-10

**Authors:** Olivia Masi Biller, Lynn S. Adler, Rebecca E. Irwin, Caitlin McAllister, Evan C. Palmer-Young

**Affiliations:** 1 Department of Biology, University of Massachusetts at Amherst, Amherst, Massachusetts, United States of America; 2 Department of Biology, Dartmouth College, Hanover, New Hampshire, United States of America; Rutgers University, UNITED STATES

## Abstract

Floral nectar contains secondary compounds with antimicrobial properties that can affect not only plant-pollinator interactions, but also interactions between pollinators and their parasites. Although recent work has shown that consumption of plant secondary compounds can reduce pollinator parasite loads, little is known about the effects of dosage or compound combinations. We used the generalist pollinator *Bombus impatiens* and its obligate gut parasite *Crithidia bombi* to study the effects of nectar chemistry on host-parasite interactions. In two experiments we tested (1) whether the secondary compounds thymol and nicotine act synergistically to reduce parasitism, and (2) whether dietary thymol concentration affects parasite resistance. In both experiments, uninfected *Bombus impatiens* were inoculated with *Crithidia* and then fed particular diet treatments for 7 days, after which infection levels were assessed. In the synergism experiment, thymol and nicotine alone and in combination did not significantly affect parasite load or host mortality. However, the thymol-nicotine combination treatment reduced log-transformed parasite counts by 30% relative to the control group (*P* = 0.08). For the experiment in which we manipulated thymol concentration, we found no significant effect of any thymol concentration on *Crithidia* load, but moderate (2 ppm) thymol concentrations incurred a near-significant increase in mortality (*P* = 0.054). Our results tentatively suggest the value of a mixed diet for host immunity, yet contrast with research on the antimicrobial activity of dietary thymol and nicotine in vertebrate and other invertebrate systems. We suggest that future research evaluate genetic variation in *Crithidia* virulence, multi-strain competition, and *Crithidia* interactions with the gut microbe community that may mediate antimicrobial activities of secondary compounds.

## Introduction

Floral nectar chemistry can shape a wide range of plant interactions with animals [1,2]. In addition to predominant chemical components such as simple sugars, water, and amino acids, floral nectar may contain a diversity of plant secondary compounds including alkaloids, glycosides, phenolics and terpenoids, which have been documented in the nectar of at least 21 plant families [1,3–7]. Nectar chemistry may reflect the ecological pressure for plants to attract and reward mutualists such as pollinators [3–5,8]. However, flowers are key areas for the growth of microbial parasites due to their warm, sugar-rich environment, and can serve as sites of parasite transmission both to plants and to insect visitors [5,9,10]. Nectar secondary compounds may also have antimicrobial activities that defend mutualist visitors against parasites transmitted at flowers [3–5].

There is broad evidence that secondary compounds have antimicrobial effects that mediate insect parasite resistance [1,9,11,12]. In insects, consumption of secondary compounds can increase host fitness and decrease the fitness of one or more parasites [13,14]. For example, consuming pyrrolizidine alkaloids (PAs) increased the resistance and survival of *Grammia incorrupta* caterpillars parasitized by *Exorista mella* flies, compared to infected caterpillars on diets without PAs [15,16]. Among pollinators, parasitized *Danaus plexippus* preferentially oviposit on *Asclepias linnaeus* that contained leaves with higher concentrations of cardenolides [17,18]. *Danaus plexippus* offspring reared on high cardenolide plants benefited from reduced parasite loads and increased longevity compared to those reared on low cardenolide plants. Thus, consumption of plant secondary compounds can provide benefits in terms of parasite resistance to animals that can tolerate these compounds.

Although the role of secondary compounds mediating protection against parasites in herbivores is well documented [14], there is a scarcity of literature regarding the antimicrobial effect of secondary compounds on pollinators, especially bees. However, several studies have suggested that these compounds could be important mediators of parasitism. In one study, natural doses of thymol and resveratrol reduced *Nosema ceranae* parasite load in *Apis mellifera*, and increased host survival [19]. Similarly, *A*. *mellifera* nurse bees infected with *N*. *ceranae* that consumed monoforal sunflower honey had a significantly lower parasite load than those fed honeydew honey [20]. Additionally, pathogen loads of *Crithidia bombi*, an obligate gut parasite of bumble bees, was reduced in *Bombus impatiens* by the consumption of gelsemine in one study [21] and by alkaloids, terpenoids, and iridoid glycosides in another [22]. The infectiousness of *C*. *bombi* may be closely related to floral traits such as nectar chemistry [23] due to the horizontal transmission of *C*. *bombi*, which occurs at flower surfaces contaminated with feces deposited by infected hosts [23–25]. In fact, *Bombus* species parasitized by *C*. *bombi* may selectively feed on plants depending on their nectar chemistry, for infected bees demonstrated a preference for diets containing the alkaloid nicotine that lowered the infection intensity [26].

Although these studies suggest that nectar chemistry plays a vital role in pollinator-parasite dynamics, several important aspects of nectar chemistry have not yet been considered. One aspect is the potential for antimicrobial synergy (i.e., non-additive effects) between multiple secondary compounds. Investigating synergisms is important for generalist *Bombus* spp., for they encounter floral chemicals as blends while foraging on different plant species in natural populations, and many plants contain multiple secondary compounds [27]. Experiments that directly tested phytochemical synergies in pollinator systems found that polyfloral sources of resin and honey increased *A*. *mellifera* resistance to parasites more than monofloral sources [20,28,29]. Literature on arthropod-herbivore systems is rich with additional examples of phytochemical synergies. For example, *G*. *incorrupta* caterpillars most effectively resisted predatory ants when consuming a mixed diet of iridoid glycosides and pyrrolizidine alkaloids, compared to hosts on single compound diets [30]. Also, two iridoid glycosides found in *Plantago* spp. synergistically increased survival and development of the specialist caterpillar *Junonia coenia* [31].

In addition, little is known about the relationship between secondary compound concentration and pollinator resistance to parasites. The concentration of chemical components in floral nectar can vary naturally between flowers on the same plant, plants of the same species, and among plant communities [5]. There are several known impacts of secondary compound concentration on pollinator behavior and survival. Multiple studies found that naturally occurring concentrations of nectar secondary compounds stimulated foraging in pollinators [8,32] and increased pollination services [32], while higher concentrations acted as feeding deterrents [8,32,33]. In terms of pollinator survival, many nectar alkaloids deterred *A*. *mellifera* above a certain concentration (50–2,000 ppm), and had toxic effects either above or below concentrations that elicit food rejection (80–2,000 ppm) [34]. For host-parasite relationships specifically, thymol and carvacrol exhibited threshold profile dose-responses against sheep and pig gut parasites [35,36]. Previous studies that manipulated nectar secondary compound presence vs. absence are valuable for establishing impacts on parasites and pollinators, but do not provide the range of potential outcomes that may occur when considering the effect of natural variation in compound concentration.

To test whether secondary compounds in nectar impact *B*. *impatiens* resistance to the gut parasite *C*. *bombi*, we conducted two experiments that investigated the potential for an alkaloid, nicotine, and a terpenoid, thymol, to affect *C*. *bombi* load and host survival. We chose these secondary compounds due to their demonstrated antimicrobial effects against pollinator parasites [19,22]. In the first experiment, we assessed whether dietary thymol and nicotine act synergistically to increase *B*. *impatiens* resistance to *C*. *bombi*, and in the second experiment, we tested whether thymol concentration mediates infection intensity. Together, these experiments tested the hypothesis that floral secondary compounds are medicinal for parasitized pollinators.

## Materials and Methods

### Study system

*Bombus impatiens* is one of the most abundant wild bee species in the eastern United States, and is commonly used in commercial agriculture due to its ability to buzz pollinate [37,38]. Queens from annual *Bombus* spp. emerge from hibernation in the spring, and found a colony of sterile workers that grows in size until mid-summer [39]. Then colonies produce queens and drones that leave the hive to mate in autumn, and inseminated queens hibernate through winter. Although *B*. *impatiens* populations are currently stable, this species is a model for studying bee-parasite interactions implicated in *Bombus* spp. declines worldwide [37,40–43].

*Crithidia bombi* is a protozoan endoparasite of *Bombus* spp. [23,40,44]. This parasite infects bees on multiple continents, with infection prevalence documented in ≤ 10% of *Bombus* spp. sampled in North America, although local infections can reach 80% [40] in the United States and 6.5–35% in Switzerland [45–48]. *B*. *impatiens* is a suitable species in which to study *C*. *bombi*, since this pollinator had the highest infection prevalence of 19 *Bombus* spp. surveyed in the eastern United States [45]. Outcomes of *C*. *bombi* infection include increased mortality in stressed hosts, impaired foraging abilities, and decreased queen fitness by 40% [49–54]. Recently, *C*. *bombi* infection was linked to *Bombus* spp. population decline [55].

Numerous experiments have established the antimicrobial properties of thymol and nicotine on parasites directly and on parasitized hosts. Pure thymol and thymol as an essential oil constituent were effective treatments against parasites that harm pollinators such as *Varroa destructor* (74,000 ppm) and *N*. *ceranae* (100 ppm), fungi such as *Candida* species (6,000 ppm), plant pathogens (1 ppm), tropical diseases (50 ppm), and several species of pathogenic bacteria (380,000 ppm) [19,56–61]. Research on the antimicrobial properties of nicotine is limited, but nicotine inhibited gram negative and gram positive bacteria (100–2,000 ppm), fungal pathogens (100–250 ppm), and parasitoids that harm invertebrates (7,000 ppm) [62–64].

The majority of these experiments found that thymol and nicotine were antimicrobial at concentrations several orders of magnitude higher than what bees naturally consume in nectar and honey. Although the concentration of nectar thymol has yet to be identified in any plant species, thymol was measured at 0.2 ppm in *Tilia europa* honey [65], while nectar nicotine occurs at concentrations of 0.1 to 5 ppm in several *Nicotiana* species [34,66,67]. Such low concentrations appear to be well within the tolerance limits of adult bees (*Apis mellifera* LD_50_: 700 ppm for thymol [68]; 2,000 ppm for nicotine [34]). However, previous work showed that naturally occurring levels of both thymol (0.2 ppm) and nicotine (2 ppm) were sufficient to reduce *C*. *bombi* loads in *B*. *impatiens* [22].

### Dietary treatments

For the thymol-nicotine synergy experiment, we tested the effects of four dietary treatments on the *C*. *bombi* load of parasitized *B*. *impatiens*: thymol (0.2 ppm), nicotine (2 ppm), their combination (0.1 ppm thymol, 1 ppm nicotine), and a control treatment (30% sucrose in deionized water). All secondary compound treatments were formulated in 30% sucrose in deionized water. A thymol stock solution was prepared by dissolving thymol to 1,000 ppm in 95% aqueous ethanol due to poor solubility in water. This stock solution was diluted in 30% sucrose solution to reach the desired treatment concentrations. The same amount of 95% ethanol was added to all treatment solutions to control for any effects of ethanol. The nicotine and thymol concentrations in the combination treatment were chosen to reflect the consumption patterns of a bee that consumed a mixed diet of nectar from different floral species comprising both compounds. The experiment contained 82 bees, 59 of which survived to dissection, with one extreme outlier removed because of an atypically high *C*. *bombi* count (Z score = 5.003).

In the thymol dose-response experiment, we measured the impact of three thymol concentrations (0.2 ppm, 2 ppm, 20 ppm) prepared with 30% sucrose in deionized water and a control treatment (30% sucrose) on *C*. *bombi* load. Thymol was dissolved to 1,000 ppm in 95% ethanol to form a stock solution, which was diluted in 30% sucrose to reach the thymol concentration of each respective treatment. Afterwards, 95% ethanol was added to the control, 0.2 ppm, and 2 ppm thymol treatments so that all solutions contained the same concentration of ethanol. The experiment contained a total of 158 bees, 133 bees of which survived to dissection.

### *B*. *impatiens* colony infection and maintenance

To establish infected *B*. *impatiens* colonies as a source of *C*. *bombi*, we collected wild bees in September, 2013 from two sites in Amherst, MA (42°23'18.81"N 72°31'21.63"W and 42°24'32.47"N 72°31'39.57"W). Gut homogenates from parasitized wild bees were fed to workers bees of commercially bred colonies (Biobest, Ontario, Canada) to spread infection throughout the colonies. Infected colonies are referred to as “source” colonies hereafter; these source colonies were complete hives that were entirely independent of the colonies that produced experimental bees. Source colonies were kept isolated from uninfected *B*. *impatiens* colonies, which are referred to as “experimental” colonies. Experimental colonies were screened weekly to confirm absence of *C*. *bombi* infection by microscopically examining gut tracts of 5 haphazardly chosen workers per colony. Four experimental colonies were used for the thymol-nicotine synergy experiment, one of which contributed only three bees and was excluded from the analysis; three colonies were used for the thymol dose-response experiment. All *B*. *impatiens* colonies were kept in darkness at room temperature, and fed multifloral pollen and 30% sucrose prepared in deionized water *ad libitum*.

### Inoculation of *B*. *impatiens* with *C*. *bombi*

To procure uninfected workers, pupal clumps were harvested weekly from uninfected experimental colonies with forceps. Pupal clumps were incubated in a Percival (Perry, IA) incubator at 30°C in darkness, and checked daily for emergence of new ‘callow’ bees. Callows were weighed and randomly assigned to treatments and isolated in individual 18.5 mL clear, plastic vials fitted with a feeding apparatus (S1 Fig). Experimental bees were fed 500 μL of 30% sucrose control solution and a 6 mg lump of multifloral pollen (Koppert Biological Systems, Howell, Michigan) for two days. On the third day, experimental bees were starved for 3–6 h before inoculation with *C*. *bombi*.

To prepare inoculum, individual gut tracts from source colony workers were removed and homogenized with 300 μL of deionized water in microcentrifuge tubes. Following a 4 h settling period, parasite cells were counted in a 10 μL aliquot of the supernatant using a Neubauer hemacytometer. A 0.02 μL volume of the gut solution was surveyed for *C*. *bombi* cells. Extracts of 2–3 gut samples were mixed and diluted to 1,200 cells/μL, then mixed with an equal volume of 50% sucrose to obtain an inoculum containing 6,000 cells/10 μL prepared in 25% sucrose, similar to the average exposure to *C*. *bombi* in the feces of infected bees [69]. After the starvation period, each experimental callow was fed a 10 μL droplet of *C*. *bombi* inoculum.

### Experimental conditions and assessment of parasite load

After inoculation, experimental bees were housed individually and fed 500 μL of treatment solution plus 6 mg of pollen daily (S1 Fig). Experimental bees were moved daily to a clean vial provisioned with fresh treatment solution and pollen, and were housed in an incubator at 30°C in darkness. Bees were dissected 7 d after infection because it was found that *C*. *bombi* infection intensity typically peaks at around this time post-inoculation [54]. During dissection, individual gut tracts were homogenized with 300 μL of deionized water in microcentrifuge tubes. After a 5 h settling period, *C*. *bombi* cell counts were performed as described for preparing inoculum. Postmortem, we measured the length of the radial cell of each bee’s right forewing as an estimate of body size for use as a covariate [70].

### Statistical analyses

Analyses were conducted using R v3.0.1 [71]. We used a linear mixed effects model [72] to assess the effect of secondary compound treatments on the response variable of *C*. *bombi* cell count. Raw cell counts (*C*. *bombi* cell count * 0.02 μL gut extract^-1^) were ln (x+1)-transformed to meet assumptions of normality and homogeneity of variance. Predictor variables included dietary treatment and experimental colony of origin as fixed effects, date of inoculation as a random effect, and radial cell length, bee mass, and number of days from bee emergence to inoculation as covariates. Covariates and interaction terms were tested for significance using Wald tests (Anova function in package “car”) [73], then sequentially removed from the final models when not significant. All bees that died before their scheduled dissection date were excluded from the *C*. *bombi* analysis. For post-hoc pairwise comparisons between treatments, we applied Tukey’s HSD tests of differences of least squared means.

We also assessed whether diet treatment affected mortality over the seven day experimental period. For the thymol-nicotine synergy experiment, exact dates of death were not recorded, so we used logistic regression to compare probability of mortality before 7 d among treatment groups. For the thymol dose-response experiment, in which time of death was recorded daily, we used a Kaplan-Meier survival analysis [74] evaluated using the Wald statistic from a Cox proportional hazards test, with thymol concentration as the predictor variable and survival hazard function as the response variable. Colony of origin was included in the model initially, but excluded from the final model because it did not explain significant variation in survival.

## Results

### Thymol-nicotine synergy experiment

**Fig 1:**
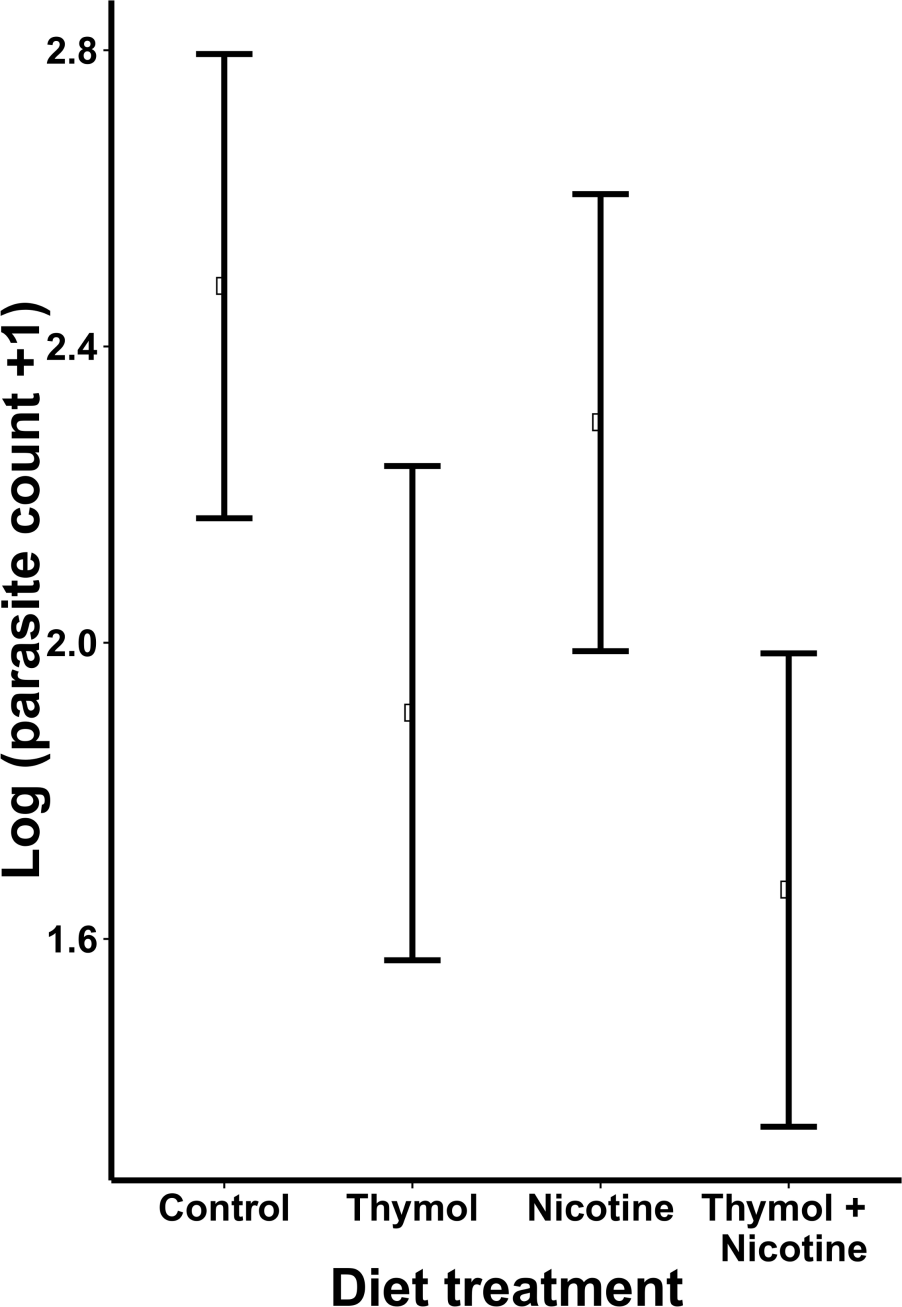
The effects of dietary thymol, nicotine and a thymol-nicotine combination on *C*. *bombi* parasitism of *B*. *impatiens*. Points indicate marginal means for each diet treatment; error bars show ± 1 standard error of the mean. Treatment had a marginally significant effect on *C*. *bombi* parasite load (*P* = 0.06), with pairwise comparisons showing a trend of reduced parasite counts in the nicotine + thymol group (*P* = 0.08).

Diet treatment had a marginally significant effect on parasite load (χ^2^ = 7.36, df = 3, *P* = 0.06, Fig 1), with the nicotine + thymol combination treatment resulting in a marginally significant reduction in parasite load compared to the control group (t_43_ = 2.44, *P* = 0.08). All other pairwise comparisons between treatment groups were not significant. Colony was a significant predictor of parasite load (χ^2^ = 9.14, df = 3, *P* = 0.03). The mortality analysis showed no significant effect of treatment on survival (χ^2^ = 1.13, df = 3, *P* = 0.77).

### Thymol dose-response experiment

**Fig 2:**
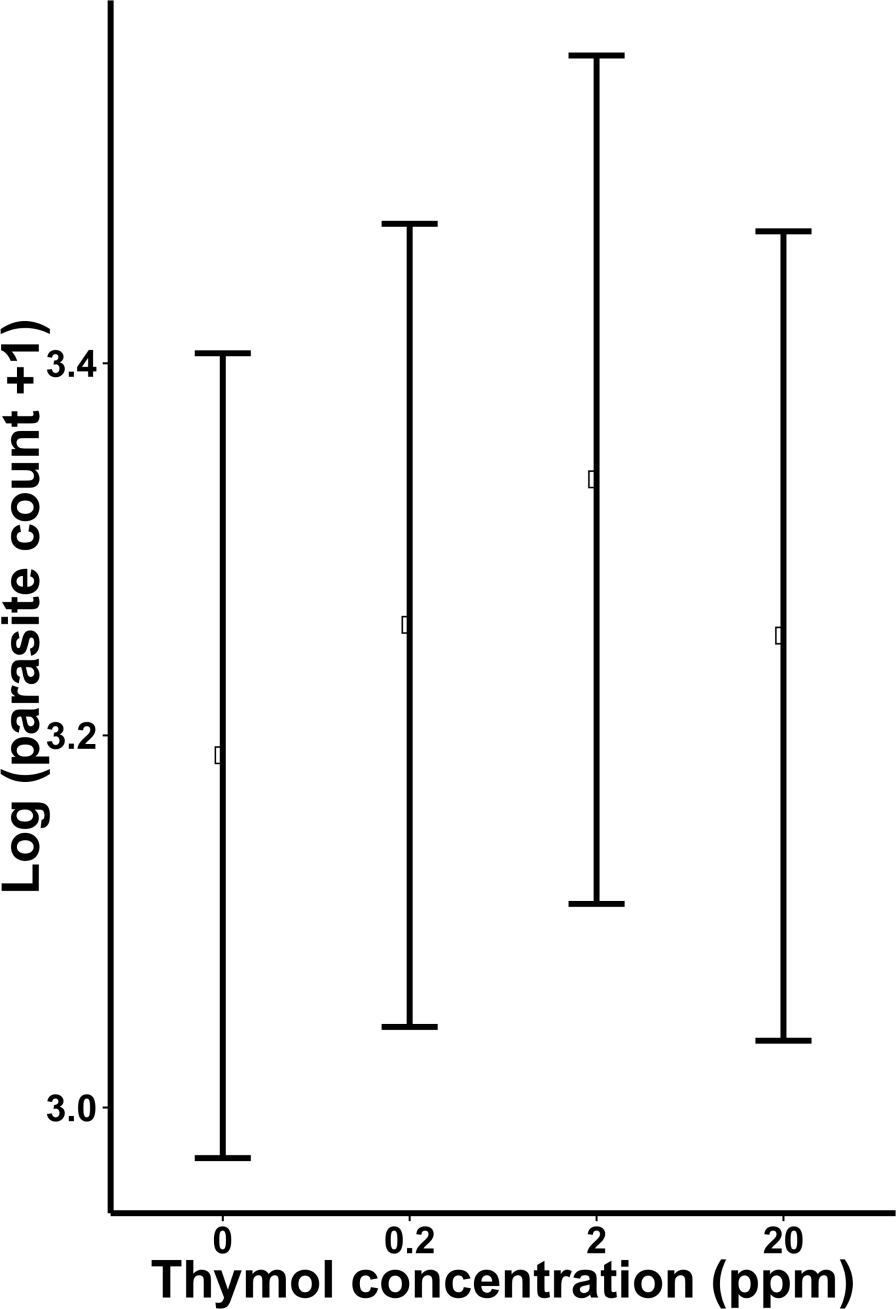
The effect of dietary thymol concentration over three orders of magnitude on *C*. *bombi* parasitism of *B*. *impatiens*. Points indicate marginal means for each diet treatment; error bars show ± 1 standard error of the mean. Dietary treatments had no significant effect on parasite loads compared to the control (0 ppm) treatment.

**Fig 3:**
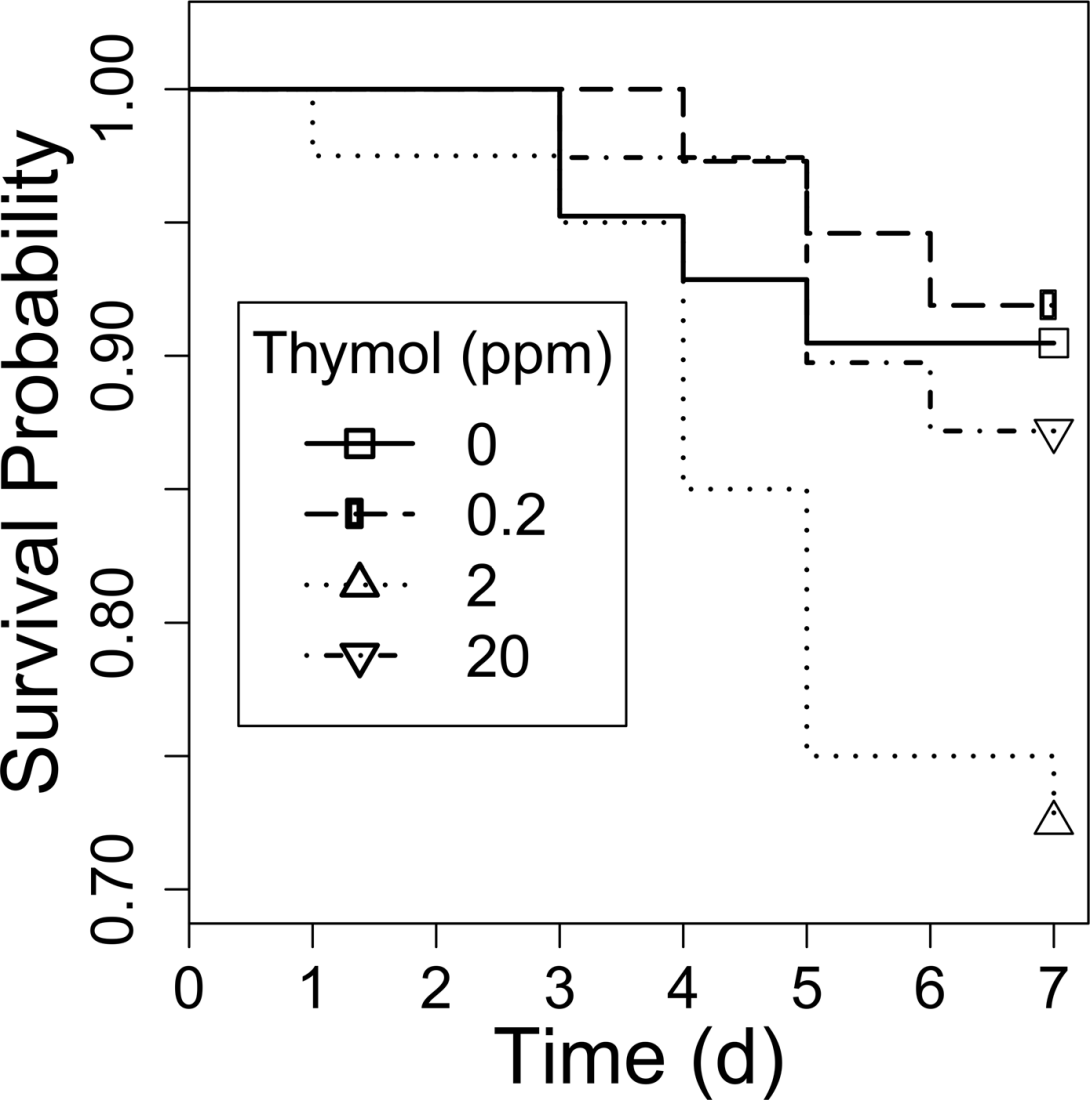
*B*. *impatiens* survival on different concentrations of dietary thymol. There was a marginally significant effect of thymol concentration on *B*. *impatiens* mortality. Pairwise comparisons showed that bees fed the 2 ppm treatment were more likely to die over the course of seven days compared to bees fed the control (0 ppm) treatment (*P* = 0.054).

Thymol concentration did not significantly affect parasite loads (χ^2^ = 0.31, df = 3, *P* = 0.96; Fig 2), nor did source colony (χ^2^ = 1.23, df = 2, *P* = 0.54). In the mortality analysis, the Cox proportional hazards test showed a marginally significant effect of diet treatment on rate of death (Wald statistic = 6.87, df = 3, *P* = 0.08; Fig 3). Pairwise comparisons suggested that bees fed the 2 ppm thymol concentration tended to have a higher probability of dying than those fed the control treatment (95% CI for odds ratio, 0.98–9.67, *P* = 0.054). In contrast, the 0.2 ppm and 20 ppm thymol treatments did not increase mortality compared to the control group (*P* > 0.05).

## Discussion

### Thymol-nicotine synergism

We observed an intriguing trend of thymol-nicotine synergy, in which the compounds in combination showed a non-significant trend of reducing *C*. *bombi* loads (*P* = 0.08). The 30% decrease in natural log-transformed parasite counts in the combination treatment corresponds to a 54% decrease in untransformed parasite load relative to the control group. The relatively small sample size in this experiment—n = 13–16 bees per diet treatment, as opposed to the n = 35 used to test the effects of gelsemine [21]—may have limited our statistical power to detect treatment effects. Our result is arguably a conservative estimate of compound interactions, for a combination of only two compounds does not realistically reflect the effects of a mixed diet on *Bombus* spp. immune status. For example, the essential oil of *Thymus zygus* aerial tissues is comprised of 39.6% thymol but also contained 36 other secondary compounds [57]. On the landscape level, *Bombus* spp. collected nectar and pollen from 33 different flowering plants in chalk grassland, and each plant may have a unique profile of floral secondary compounds [75]. To further assess secondary compound interactions, it would be useful to test effects of wild *Bombus* spp. diet breadth and corresponding secondary compound diversity on parasite resistance in natural plant communities.

### Nicotine and increasing thymol concentration were not antimicrobial

Parasite load was unaffected by consumption of nicotine and consumption of thymol over three orders of magnitude. Our results contrast with prior results from our lab, in which thymol and nicotine reduced *C*. *bombi* loads [22]; these experiments were conducted in our laboratory using protocols identical to those described here. The main differences between our experiments and the previous work are that we used a *C*. *bombi* strain collected in a different year (but from the same sites), and different *B*. *impatiens* colonies (but from the same supplier) [22]. We propose a number of non-exclusive mechanisms to explain the inconsistent effects of nicotine and thymol consumption on *C*. *bombi* parasitism.

Our divergent findings could be attributed to genetic variation in parasite resistance to thymol and nicotine. Results from another host-trypanosome system are consistent with this hypothesis. Strains of *Plasmodium falciparum*, the agent of malaria, evolved resistance to most antimalarial chemical classes in common use; specific resistance mutations occurred spontaneously in the *P*. *falciparum* genome independent of drug use, and these molecular markers can be used to assess antimalarial susceptibility [76,77]. *C*. *bombi* populations may have a similar tendency to evolve, for *Bombus* spp. colonies are parasitized by diverse, genetically distinct strains of *C*. *bombi* that differ in their levels of infectiousness [78–81]. Therefore, the *C*. *bombi* strains used in our experiment could have harbored mutations that conferred resistance to secondary compounds. In addition, different *Crithidia* species have been found to co-occur throughout the range of *C*. *bombi* [82]. Hence, it is possible that the parasites in our source colonies included different species from those used in previous studies that could not be distinguished under the microscope; these different species could vary in tolerance to secondary compounds. Future research should test for heterogeneous *in vivo* effects of secondary compounds on host resistance. To do so, *C*. *bombi* strains (singly or in combination) that differ in their susceptibility to secondary compounds should be used to infect *Bombus* spp. workers fed secondary compounds.

The effects of secondary compounds might vary depending on colony genotype as well as parasite genotype. The significant variation in parasite load across source colonies in the thymol-nicotine synergy experiment suggests that host genotypes differed in their susceptibility to the parasite, despite the fact that all colonies were obtained from the same commercial rearing facility. This result is consistent with previous work, which indicated that the coevolution of *Bombus* hosts and their *C*. *bombi* parasites involves reciprocal natural selection [78,80,81]. Although we did not find any interactions between colony and treatment within this experiment, it comprised only four colony genotypes. In contrast, previous work has indicated that interactions between *B*. *impatiens* colony genotype, parasite genotype, and environmental factors such as diet led to heterogeneity in infection outcomes in multiple experiments [78,83]. Future experiments should survey a broader range of colonies, including those reared from genetically diverse wild queens, to determine how colony genotype to interacts with secondary compound treatment to determine *C*. *bombi* load.

Another explanation for why thymol and nicotine lacked antimicrobial properties in our experiments is that mixed-genotype *C*. *bombi* infections may have impacted the effects of secondary compounds. Infections in our lab colonies were established by repeatedly inoculating their worker bees with gut homogenates of heavily parasitized wild bees. In surveys of wild *Bombus* spp., over one half of workers infected with *C*. *bombi* carried multiple strains, and strain diversity was positively correlated with infection intensity [84–86]. Hence, we expect that our experimental inoculum comprised multiple *C*. *bombi* genotypes. In baculovirus infections, diverse infections had greater pathogenicity, with the pathogenicity of each strain differing according to both infection diversity and host diet [87]. We speculate that the composition and diversity of our *C*. *bombi* strains differed from those used in previous experiments, and that interactions among the different strains may mediate the effects of thymol and nicotine. This idea could be tested by comparing the sensitivity of single- and mixed-genotype infections to secondary compounds.

Finally, specific gut microbe species may shape the antimicrobial activity of secondary compounds. The inoculum used in our experiments was derived from gut homogenates that contained the full complement of microbiota of infected bees. In other experiments, the origin of *Bombus spp*. gut microbiota was an important predictor of *C*. *bombi* infection outcome [80,88,89]. Host variation in *C*. *bombi* susceptibility to secondary compounds may be in part due to presence or absence of particular gut-dwelling species [89]. To study how gut microbiota may influence secondary compound activities, the effects of secondary compound consumption could be compared between infected bees that have been colonized with different endogenous microbiota. Such experiments may help to elucidate how, and in what contexts, secondary compounds like nicotine and thymol exert antiparasitic effects.

### Moderate thymol dose reduced host survival

Research on the effects of thymol and nicotine on bee colony development and survival is scarce [22,67,90], but supports our finding that neither compound greatly impacts mortality of adult bees at concentrations that occur naturally in nectar, at least under controlled conditions. Although both compounds may be toxic to larvae at concentrations higher than the natural range—such as 50 ppm nicotine and the 250,000 ppm thymol used in the *V*. *destructor* treatment Apiguard (Vita, Hants, United Kingdom) [56,67]—our thymol (0.1–20 ppm) and nicotine (1–2 ppm) treatment concentrations were far below those associated with mortality in adult honey bees, where the 6-day LD_50_ was 700 ppm for thymol [68] and the 2-day LD_50_ was 2,000 ppm for nicotine [34]. Therefore, we doubt that the mortality in the 2 ppm thymol treatment reflects direct toxicity of thymol at this concentration, particularly because the much stronger 20 ppm treatment had no effect on mortality.

We postulate that the 2 ppm thymol concentration stimulated foraging behavior, as has been shown for other monoterpenes [91], and that these behavioral changes may have increased mortality by two mechanisms: increased physical trauma and water intoxication. First, foraging behavior could have increased physical trauma experienced by bees while attempting to fly and during the daily changing of their feeding apparatus. Experimental bees were often observed crashing into the transparent walls of the vial as they attempted to initiate flight. In addition, bees that held tenaciously to their vials’ feeding wicks (S1 Fig) had to be mechanically disturbed by firmly tapping the vial against the lab bench before the bees could be transferred to clean vials. Although these procedures were performed as gently as possible, the cumulative effects of such manipulation could have increased mortality over the weeklong experimental period.

Second, increased consumption of the 2 ppm treatment solution may have resulted in electrolyte depletion and water intoxication. Although we unfortunately did not measure nectar consumption, previous work in both *B*. *impatiens* and *A*. *mellifera* showed a relationship between consumption patterns and compound concentration, with low concentrations of secondary compounds acting as feeding stimulants, but high concentrations acting as deterrents [8,32]. Similarly, the 2 ppm thymol treatment may have stimulated consumption, whereas the 20 ppm treatment may have curbed consumption. Following this logic, bees consuming 2 ppm thymol could have ingested a relative excess of treatment solution, which was dissolved in deionized water and therefore lacked essential ions. Bees in natural situations consume electrolytes in nectar and pollen, such as Na^+^, K^+^, Ca^2+^, Mg^2+^ and Cl^-^ ions [92–94]. Although nectar-feeding insects are adapted to conserve electrolytes and process water loads, they do excrete measurable amounts of ions [95,96]. Also, prior studies indicate that metabolic water production exceeded evaporative water losses in *Bombus* spp [96,97]. Therefore, it was hypothesized that *Bombus* spp. have difficulty dealing with excess water loads, which may lead to impaired osmoregulation and increased likelihood of death [97]. This may be similar to water intoxication in humans, which can occur due to excess water load and electrolyte depletion [98,99]. Feeding preference tests assessing the impact of thymol solution concentration on consumption rates, evaporative water loss, and electrolyte levels are necessary to test this hypothesis of water toxicity.

In conclusion, the results of both experiments showed that nicotine and thymol were neither significantly medicinal nor toxic at the concentrations and combinations tested. However, the thymol-nicotine combination treatment tended to lower parasite load compared to the control, suggesting potential benefits of mixed diets for host immunity. Colony of origin was a significant factor in the thymol-nicotine experiment, indicating the potential relevance of genetic variation for host resistance and parasite virulence. Additionally, the 2 ppm thymol treatment resulted in a near-significant increase in host mortality, perhaps reflecting the effects of electrolyte depletion. Our findings coincide with literature that showed that thymol and nicotine were not toxic at concentrations found in nectar and honey [8,22,90], yet contrast with literature that demonstrated the antimicrobial properties of thymol and nicotine at higher doses and in other contexts [19,22,57–63,100]. Future research on the impact of diet breadth, host-parasite genetic variation, and gut microbe communities on infection outcome may reconcile our findings with results from previous work. Further analysis of the effects of *Bombus* spp. diet on immunity is necessary to assess the conditions under which floral secondary compounds can defend pollinators from microbial pathogens.

## Supporting Information

S1 Fig Housing and feeding apparatus for experimental bees. An 18.5 mL vial was used to house each experimental bee, outfitted with a feeding apparatus composed of a 2 mL microcentrifuge and 0.6 cm cotton wick adhered to a snap cap lid that was punctured with ventilation holes. Each feeding apparatus’ microcentrifuge tube was provisioned 500 μL of treatment solution daily.
(PDF)
