# Supplementary figures and images for: Possible Synergistic Effects of Thymol and Nicotine against Crithidia bombi Parasitism in Bumble Bees
Source: PLoS One. 2015 Dec 10;10(12):e0144668. doi: 10.1371/journal.pone.0144668 (PMC4686078; doi:10.1371/journal.pone.0144668)

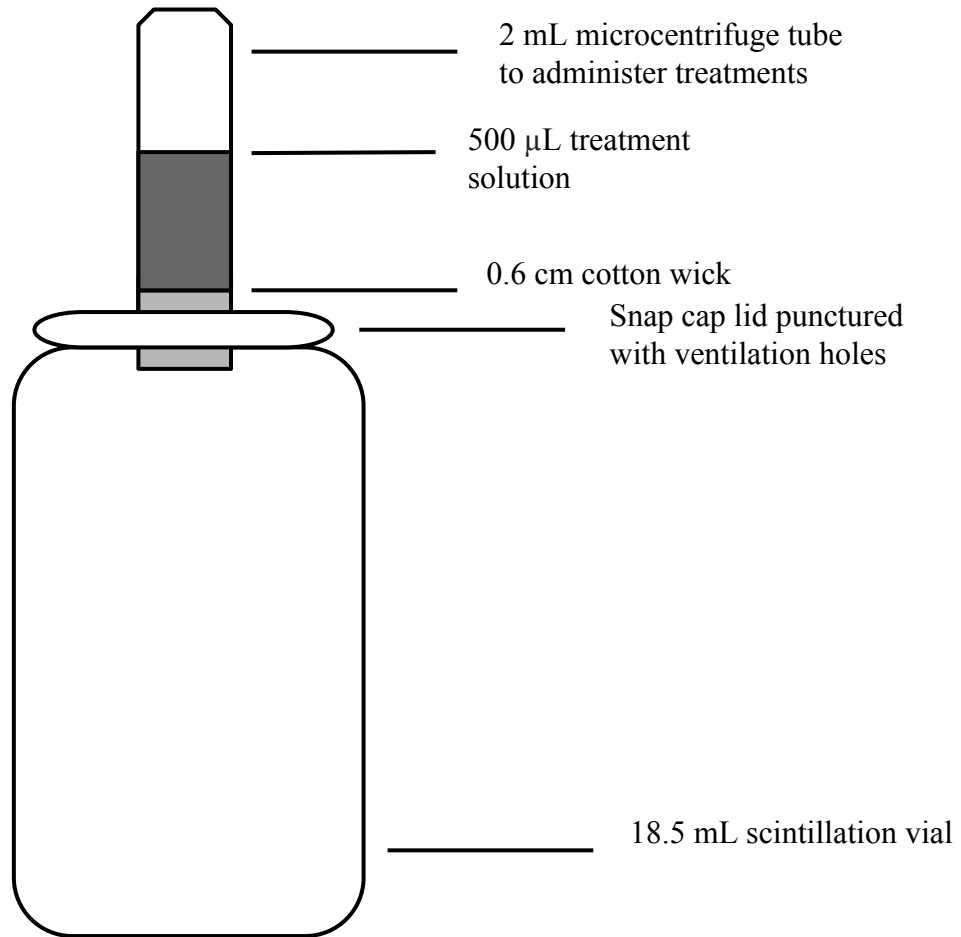

Supplement: S1 Fig — An 18.5 mL vial was used to house each experimental bee, outfitted with a feeding apparatus composed of a 2 mL microcentrifuge and 0.6 cm cotton wick adhered to a snap cap lid that was punctured with ventilation holes. Each feeding apparatus’ microcentrifuge tube was provisioned 500 μL of treatment solution daily. (PDF) [file pone.0144668.s001.pdf]
